# High spatial resolution of the rotational sensor array in the explosion sources localization

**DOI:** 10.1038/s41598-026-50391-8

**Published:** 2026-04-29

**Authors:** Anna T. Kurzych, Leszek R. Jaroszewicz, Grzegorz Lizurek, Bartosz Sakowicz, Michał Dudek, Karol Konarski, Paweł Zinówko

**Affiliations:** 1 Military University of Technology, Warsaw, 00-908 Poland; 2 Elproma Electronics Ltd., Czosnów, 05-152 Poland; 3 Institute of Geophysics, Polish Academy of Sciences, Warsaw, 01-452 Poland; 4 Lodz University of Technology, Łódź, 93-005 Poland

**Keywords:** Rotational sensor, Vibration detection, Hypocenter localization, Sensors array, Engineering, Mathematics and computing, Physics

## Abstract

In the face of increasing security requirements, threat identification requires technology with uncompromising sensitivity and responsiveness. This article presents a sensor with enhanced sensitivity, designed to detect rotational ground movement. Its physical basis on the Sagnac effect provides an absolute measurement of rotation. From a geotechnical and engineering perspective, the configuration of several such sensors enables precise determination of the epicentral coordinates of seismic events and identification of local material degradation zones, where differences in signal transit time and amplitude between individual network nodes provide the basis for mapping discontinuities in the studied medium. The primary goal of the presented research was to verify the feasibility of creating an integrated sensor array capable of precisely monitoring a defined area. Using highly correlated sensors with time synchronization of the order of 10 ns we obtained signals from artificial disturbances. The time-of-arrival analysis of recorded sensor signals successfully demonstrated the ability to quickly determine the locations of explosions. The presented solution offers new perspectives for early warning systems, providing not only threat detection but also accurate spatial positioning of threats using highly sensitive rotational sensors.

**Supplementary Information:**

The online version contains supplementary material available at 10.1038/s41598-026-50391-8.

## Introduction

Earthquake-induced tremors result not only in vertical and horizontal displacements of the Earth but also in rotational motion. For a comprehensive understanding of seismic sources and wavefields, the literature on the subject posits the need for simultaneous measurement of linear and rotational motion^1,2^. Yet, the pursuit of this endeavor has been hampered by major technical difficulties in detecting rotational motions with sufficient sensitivity. Consequently, capturing the complete range of ground movement remains an open challenge. Building on promising preliminary research from recent years, this paper seeks to advance an instrumentation strategy designed to address new applications of rotational sensors.

In seismology, rotational motion is usually expressed in the framework of the linear elasticity theory, assuming infinitesimal deformation^3^. In a linearly elastic medium, the displacement *u* of a point *x* is related to the neighboring point *x + δx* by the following relation^4^:

1 $$u(x+\delta x)=u(x)+\varepsilon \delta x+\omega \times \delta x$$

where *ε* is the stress tensor and

2 $$\omega =\frac{1}{2}\nabla \times u(x)$$

is a pseudovector representing an infinitesimal angle of rigid rotation generated by a perturbation. Then three components of rotation about *X*,* Y*, and *Z* axes for infinitesimal motions are expressed by:

3 $$\begin{gathered} {\omega _x}=\frac{1}{2}\left( {\partial {u_z}/\partial y - \partial {u_y}/\partial z} \right) \hfill \\ {\omega _y}=\frac{1}{2}\left( {\partial {u_x}/\partial z - \partial {u_z}/\partial x} \right) \hfill \\ {\omega _z}=\frac{1}{2}\left( {\partial {u_y}/\partial x - \partial {u_x}/\partial y} \right) \hfill \\ \end{gathered}$$

The integration of 6DOF (six degrees of freedom) methodology into the analysis of ground dynamics represents a significant research breakthrough, transcending conventional seismology based solely on translational components. The recording of three orthogonal axes of rotation, combined with traditional acceleration measurements along the *X*, *Y*, and *Z* axes, allows for the complete reconstruction of the strain tensor, opening up innovative possibilities for the precise localization and characterization of disturbances in the geological medium and engineering structures^5,6^. In the geophysical dimension, the complete 6DOF information enables the direct estimation of the phase velocity of seismic waves at a single measurement point, drastically increasing the accuracy of hypocenter location, and knowledge about rotational movements give opportunity to identify the wave’s direction of arrival without the need for extensive and costly seismic networks^5–7^.

In this paper, we present the new application of FORS (Fiber-Optic Rotational Seismograph) for mechanical events mapping. The experiment implemented a measurement system comprising four independent FORS sensors, integrated via high-precision time-synchronization protocols, achieving a time synchronization of 10 ns. This rigorous synchronization regime, together with the high correlation among sensors, was essential for reliable recording of shock-wave dynamics generated by controlled explosive detonations, thereby maintaining full coherence of the recorded signals. The conducted studies provided strong support for the proposed research thesis, demonstrating that time analysis of records obtained from individual measurement units enables precise estimation of the wave arrival vector and effective localization of the perturbation source.

The paper describes the technical specifications and configuration of the instrumentation used in the research process, providing an introduction to the core of the publication: the presentation and multidimensional analysis of the results obtained during the conducted field experiments. It focuses on the interpretation of spatiotemporal data obtained from a system of four synchronized measurement units, demonstrating their ability to precisely locate the sources of dynamic disturbances. The discussion concludes with a summary of the effectiveness of the employed methodology and indicates the potential for implementing FORS technology in modern geotechnical monitoring.

### Fiber optic seismograph (FOS) construction

A fiber-optic gyroscope (FOG) technology has been known for over four decades, and its continuous development has made it one of the most promising solutions in the field of rotation sensors. FOGs are based on the Sagnac effect^8,9^, which causes a phase shift between two counter-propagating light beams when their optical path is rotated.

The presented and applied in this paper sensor FOS6 (Fiber Optic Seismograph) is a sixth generation of FORS family which historical evaluation has started in 2004^10^. The main differences between the previous systems (Fibre-Optic System for Rotational Events and Phenomena Monitoring (FOSREM), types FOS1-5)) and recent (FORS, type FOS6) is the electronic part and number of optical coils. FOSs1-5 are single-axis sensors^11^. FOSs1-4 are constructed according to an open-loop architecture with a digital data processing^12^ while type FOS5^11^ and FOS6^13^ are performed by a closed-loop configuration based on the compensatory phase measurement method. The evolution of mechanical configurations and control systems has led to significant increases in system efficiency, manifesting in the transition from simple single-axis structures to advanced three-axis configurations (FORS, type FOS6), enabling precise operation in the full spatial coordinate system. Unlike previous generation solutions, which were based on open-loop control, modern systems implement feedback mechanisms. Thanks to the use of closed-loop control, modern systems achieve significantly higher dynamics and stable frequency response, which is crucial in processes requiring high speed and precision.

**Fig. 1:**
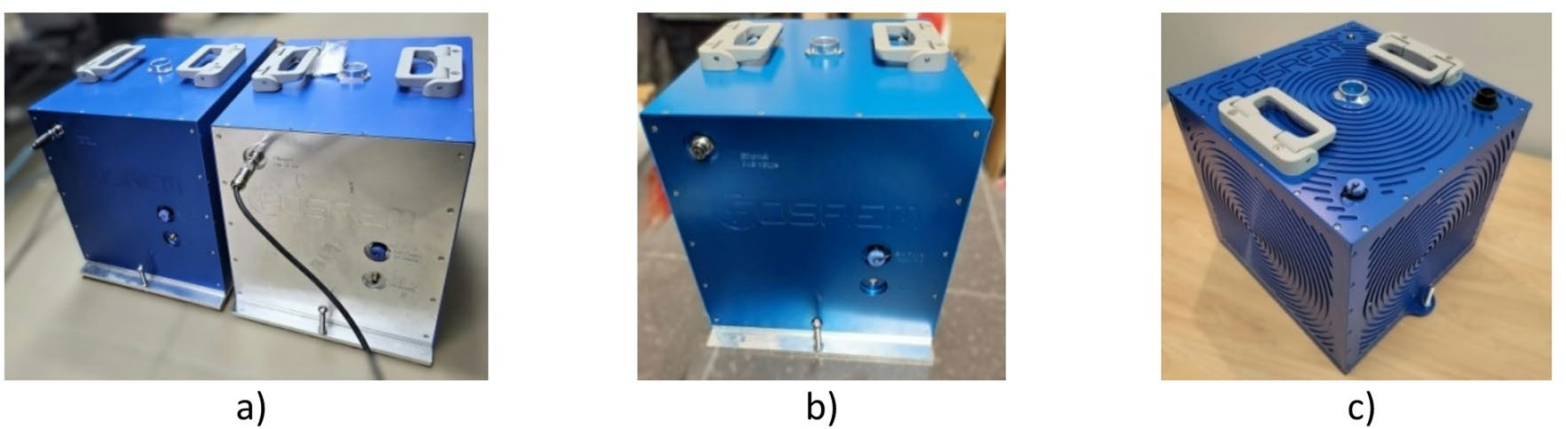
The general view of FOS6s applied in the field: (**a**) FOS6-01/-02, (**b**) FOS6-03, (**c**) FOS6-04.

The presented in this paper FOS6 consists of three independent single-axis subsystems based on the FOG structure. The primary feedback loop uses an ASE (Amplified Spontaneous Emission) light source that feeds the three axes via a 1 × 3 fiber-optic splitter. In each axis, the light is split into two waves that interfere and generate a signal proportional to the phase difference, which is converted to current by an APD (Avalanche PhotoDiode). To detect the rotation direction and increase sensitivity, the phase of the waves is modulated using an MIOC (Multi-functional Integrated Optical Circuit) according to a closed-loop approach. The system incorporates APD current regulation via a secondary feedback loop to ensure stable operation. FOS construction has been presented in broad terms^14,15^. Here, we present the last noise performance investigation and its new hermetically sealed construction. The four FOS6s shown in Fig. 1 were employed in the experimental research described in the next paragraph. Technically, these are the same devices with identical internal construction. They differ only in design: the first two variants are a matter of color choice (blue or silver, Fig. 1a, b), while the latter features additional fins (Fig. 1c). These fins serve not only an aesthetic purpose but also serve to improve heat dissipation.

The main parameters of the FOS6-01/02/03/04 are listed in Table 1. The noise parameters were determined theoretically and experimentally. ARW (Angle Random Walk) has been determined by multiplication of noise contributor expressed in µrad/√Hz and rotation (*Ω*_µ_), leading to 1 µrad of phase difference according to the formulas^16,17^:

4 $$\:\mathrm{A}\mathrm{R}\mathrm{W}\mathrm{=}{\varOmega\:}_{\mu\:}\times\:noise$$

5 $$\:{\varOmega\:}_{\mu\:}\mathrm{=}\frac{\lambda\:}{2\pi\:}\frac{c}{4A}$$

where: *λ* – wavelength of optical source, *c* – speed of light, *A* – enclosed area of sensor. The assumed noise is connected with shot noise, which is the only noise contributor inevitable in FOG^18^.

To reliably assess the stochastic parameters of the FOS6, experimental data were collected under controlled environmental conditions during nighttime sessions. This time of day was chosen to minimize the impact of anthropogenic vibroacoustic interference and thermal fluctuations, thereby maintaining a high SNR (Signal to Noise Ratio) and ensuring the stationarity of error processes over a short time. The raw measurement data were subjected to statistical processing using the ARMAV (Autonomous Regression Method for Allan Variance)^19^. This method is a modern alternative to the classical Allan variance analysis, based on time-series modeling using autoregressive and moving-average processes. ARMAV enables more precise decomposition of individual noise components, even with relatively short data sets. Using the parametric approach in ARMAV enabled higher spectral resolution and greater estimator stability than nonparametric methods. This allowed for the precise isolation of the sensor’s asymptotic characteristics and the identification of the point of minimum variance, which defines the BI (Bias Instability). Table 1 shows the theoretical and experimental values of ARW and BI determined for each axis of four FOS6s.

**Table 1:** The main parameters of the FOS6s determined theoretically and experimentally.

Unit/Axis	Coil length [m]	Enclosed area [m^2^]	ARW [nrad/s/√Hz] Theoretically / Experimentally	BI [nrad/s]
FOS6-01				
X	6 170	386	6.24 / 35	10
Y	6 286	393	6.12 / 39	13
Z	5 994	375	6.42 / 39	14
FOS6-02				
X	6 307	394	6.10 / 45	15
Y	6 012	376	6.40 / 47	25
Z	6 068	379	6.34 / 53	28
FOS6-03				
X	6 246	390	6.16 / 35	12
Y	6 231	389	6.18 / 40	14
Z	6 482	405	5.94 / 37	7
FOS6-04				
X	6 030	377	6.38 / 45	7
Y	5 837	365	6.59 / 40	7
Z	6 459	404	5.96 / 48	10

The theoretical values of ARW fluctuate within a range from 5.94 nrad/s/√Hz (for the enclosed area of 405 m^2^) to 6.59 nrad/s/√Hz (for the enclosed area of 365 m^2^), the experimental results are one order higher and stable, ranging from 35 nrad/s/√Hz (for FOS6-01, X –axis and FOS6-03, X –axis) to 53 nrad/s/√Hz (FOS6-02, Z –axis).

The systematic discrepancy between theoretical and experimental ARW values observed during the study is demonstrated by the fact that the experimental data values exceed the model predictions by nearly an order of magnitude. This significant difference is not due to sensor errors or calibration issues but is a direct consequence of the unfavorable environmental conditions under which the data were acquired. The experiment was conducted in a laboratory in Warsaw, Poland, in a highly urbanized area, which resulted in the constant presence of broadband vibroacoustic disturbances. The stochastic background noise present in the environment, generated primarily by transportation infrastructure and industrial activity, introduced additional spectral components into the measurement system, which, when superimposed on the useful signal, degraded the SNR.

The correlation between individual sensor signal recordings is a fundamental condition that determines the effectiveness of localization processes and the precise identification of vibration source parameters. High data consistency in a multi-channel system is essential for the accurate implementation of modal analysis algorithms and triangulation methods. In this context, work^13^ rigorously evaluated the Pearson linear correlation coefficient determined for time series recorded by autonomous FOS6s. Statistical estimation demonstrated that the analyzed signals exhibited an extremely high degree of coherence, with a correlation coefficient oscillating around 99%, confirming the high fidelity of the vibration field reproduction by FOS6s. This signal convergence remained at a constant, high level regardless of the dynamic excitation characteristics. A high correlation coefficient was observed for both medium-amplitude excitations (0.25 rad/s) and rapidly changing states (100 Hz) maintaining strong correlation of results even with extremely different dynamics and frequencies of input signals. A particularly significant result is the high correlation observed when registering weak rotational disturbances (amplitude 0.5 mrad/s), demonstrating the exceptional sensitivity of the sensors and the low noise of the system^13^. Such high metrological precision in detecting low-energy signals enables effective separation of useful diagnostic information from the stochastic background, thereby enabling a full characterization of the dynamic phenomena in the array system under study.

### Experiment description and data analysis

**Fig. 2:**
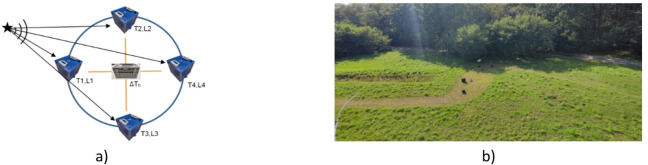
Experiment overview: (**a**) general schema of the method of the source of vibration locating; (**b**) test field of FOS6s installation to validate their application.

The experiment was conducted to validate the feasibility of localizing a vibration source. The method is based on determining the relationship between the time signatures of the recorded disturbance and the topology of the sensor arrangement in the FOS6 matrix (Fig. 2), as described in the Patent Application to the Polish Patent Office (P.450532 dated 11.12.2024).

**Fig. 3:**
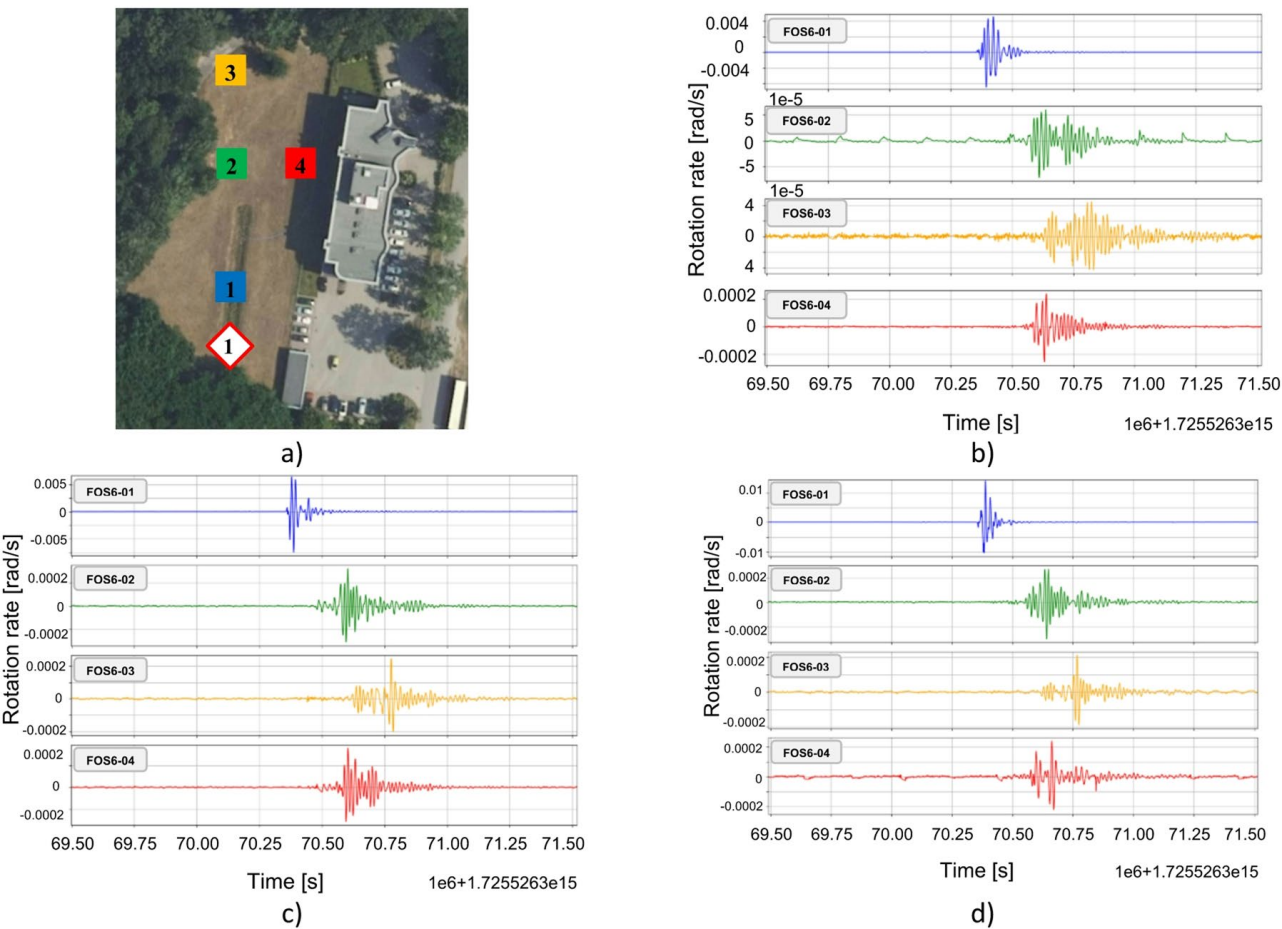
Data recorded during the explosion no. 1 by FOS6-01/02/03/04: (**a**) locations of the sensors and explosion, (**b**) signals for the Z-axis (vertical), (**c**) signals for the X-axis (East – West), (**d**) signals for the Y-axis (North – South).

**Fig. 4:**
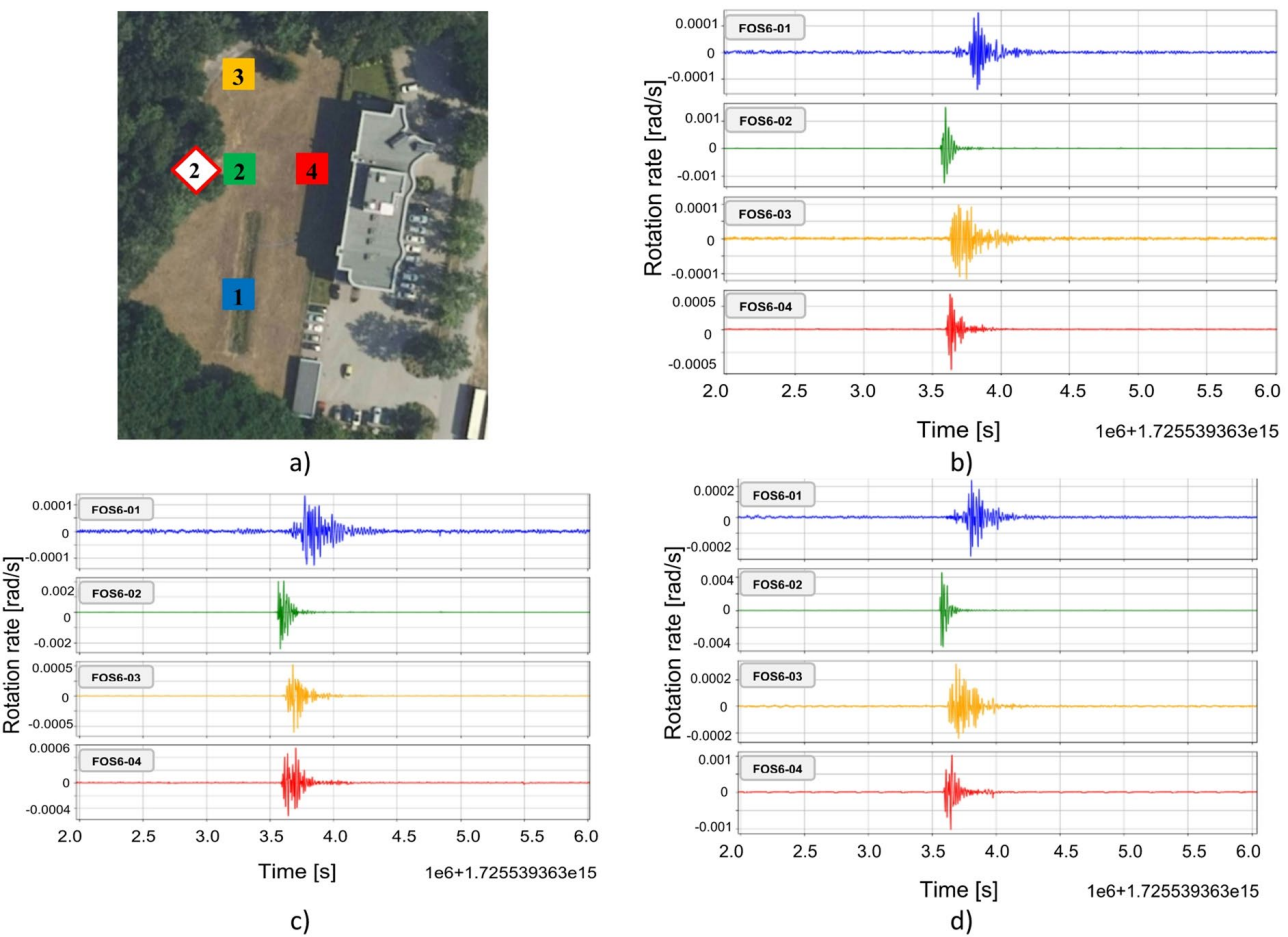
Data recorded during the explosion no. 2 by FOS6-01/02/03/04: (**a**) locations of the sensors and explosion, (**b**) signals for the Z-axis (vertical), (**c**) signals for the X-axis (East – West), (**d**) signals for the Y-axis (North – South).

**Fig. 5:**
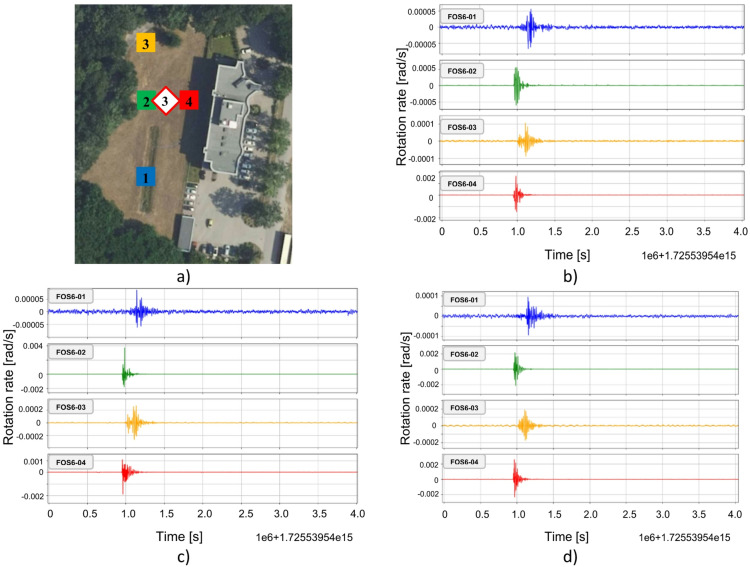
Data recorded during the explosion no. 3 by FOS6-01/02/03/04: (**a**) locations of the sensors and explosion, (**b**) signals for the Z-axis (vertical), (**c**) signals for the X-axis (East – West), (**d**) signals for the Y-axis (North – South).

**Fig. 6:**
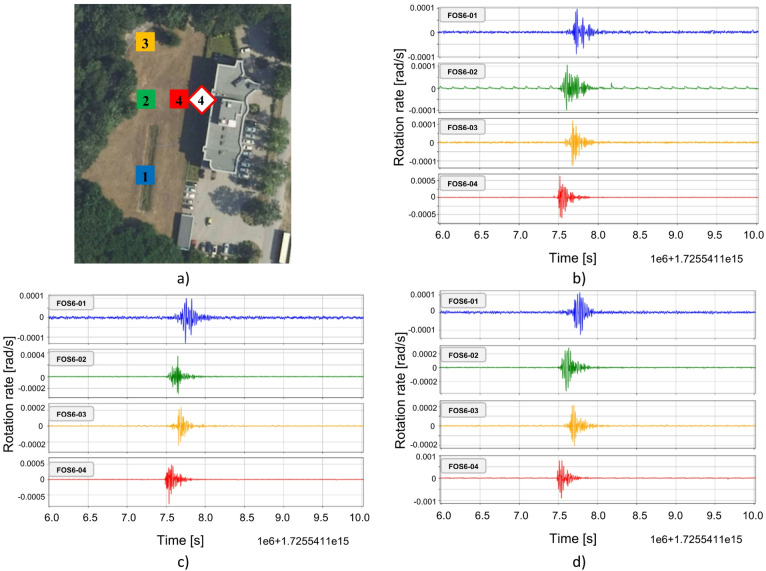
Data recorded during the explosion no. 3 by FOS6-01/02/03/04: (**a**) locations of the sensors and explosion, (**b**) signals for the Z-axis (vertical), (**c**) signals for the X-axis (East – West), (**d**) signals for the Y-axis (North – South).

In order to verify the possibility of applying FOS6s to the above mentioned method the series of experiment have been carried out in the field next to the company Elproma Electronics Ltd., Czosnów, Poland (Fig. 2b). The presented dataset has been recorded by four FOS6s during four controlled energetic events initiated under specific boundary conditions. The experiment was based on the system: explosive charge – transmission medium (water) – shielding (pipe) – soil medium. The charges were placed in rigid pipes, which were then deposited in the ground at a depth of h = 0.5 m (measured from ground level to the pipe axis). This depth classifies the explosion as a shallow explosion, in which the interaction of the shock wave with the free ground surface and the formation of the discharge funnel play a significant role. The distances between the sensors were the following (see Fig. 3): FOS6-01 (blue square) – FOS6-02 (green square) approx. 45 m; FOS6-02 – FOS6-04 (red square) approx. 12 m; FOS2 – FOS3 (yellow square) approx. 32 m. Each explosion location has been marked in Figs. 3, 4, 5 and 6 as a white rhombus with a red edge. The figures showing the FOS6s location and the specific explosion location are presented alongside the registered signals to facilitate interpretation of the data. Moreover, the recorded signals are color-coded to indicate the sensor color on the map along. The orientation of the sensor axes was pre-determined relative to geographic directions (X: East – West; Y: North – South; Z: vertical) using a magnetic compass during field installation of the devices. Next, precise orientation of the axes relative to geographic North was achieved using the gyrocompass method. Nighttime recordings of the resting signals were used to extract the Earth’s angular velocity component on the device’s horizontal axes. This allowed for high-precision determination of the installation’s azimuth and simultaneous calibration of the static bias by comparing the measured values with a theoretical model of Earth’s rotation for a given latitude.

In Table 2, the parameters of the recorded signals during explosion no. 1 (simplified timestamp: 8:53 a.m. UTC) for particular sensors and axes are presented. As one can see, the explosion was first recorded by FOS6-01, which is consistent with its location. Analysis of the signal amplitude parameters (Table 2) showed that sensor FOS6-01 recorded the signal with the highest values of the maximum absolute signal amplitude (A_max_), reaching, respectively: 4.12 mrad/s for the Z-axis, 7.53 mrad/s for the X-axis, and a dominant value of 14.01 mrad/s for the Y-axis. Such a pronounced amplitude gradient with respect to the remaining measurement units is a direct function of the distance from the shock wave center and confirms high sensitivity of the device in the near field. The chronometry of the recorded waveforms shows full consistency with the geometric arrangement of the measurement network relative to the event epicenter. Signal initiation in the sensor FOS6-01, located closest to the source, occurred at 08:53:13:11. As the wave propagation distance increased, a delay in recording by subsequent nodes was noted, up to sensor FOS6-03 (the most distant), which recorded the disturbance at 08:53:13:24. The maximum difference in the time of signal arrival was determined for the Z-axis between sensors FOS6-01 and FOS6-03 equaling 264.420 ms.

**Table 2:** Parameters of the recorded signals during explosion no. 1 for particular axis of the sensors FOS6-01/02/03/04.

Unit	Axis	Arrival of the event signal [h: min: s]	End of the event signal [h: min: s]	Event duration time [ms]	Time difference of the event signal arrival [ms] Δt_FOS6−02/−01_	A_max_ [mrad/s]
FOS6-01	Z	08:53:13.109409	08:53:13.319193	209.784	122.859	4.12
	X	08:53:13.110445	08:53:13.317069	206.624	117.876	7.53
	Y	08:53:13.109150	08:53:13.331569	222.419	122.329	14.01
					Δt_FOS6−03/−01_	
FOS6-02	Z	08:53:13.232268	08:53:13.601714	369.446	264.420	0.07
	X	08:53:13.228321	08:53:13.577242	348.921	260.532	0.32
	Y	08:53:13.231479	08:53:13.713186	481.707	260.876	0.30
					Δt_FOS6−04/−01_	
FOS6-03	Z	08:53:13.373829	08:53:13.713186	339.357	133.254	0.04
	X	08:53:13.370977	08:53:13.723642	352.665	136.304	0.25
	Y	08:53:13.370026	08:53:13.735049	365.023	135.848	0.21
					Δt_FOS6−03/−02_	
FOS6-04	Z	08:53:13.242663	08:53:13.73479	492.127	141.561	0.03
	X	08:53:13.246749	08:53:13.731288	484.539	142.656	0.36
	Y	08:53:13.244998	08:53:13.711439	466.441	138.547	0.24

A_max_ – maximal absolute signal amplitude; Δt_FOS6−0X/−0Y_ – time difference of signal arrival for given axis between FOS6-0X and FOS6-0Y.

FOS6-02 and FOS6-4 were located in the immediate vicinity of the explosion initiation point no. 2 (Fig. 4), which translated into the lowest time delay values relative to the detonation moment during data acquisition.

This proximity not only determines the order in which the shock wave front is recorded by individual array elements, but also contributes to minimal dissipation of signal energy, which is manifested by a high SNR in these specific measurement channels. Analysis of the signal chronometry (Table 3) confirmed that sensor FOS6-02 was the first to register the shock wave front, which defines it as the reference node for event no. 2. Analysis of the difference in signal arrival time between sensors FOS6-02 and FOS-04 showed high consistency across the orthogonal domains, with values of 22.815 ms (Z-axis), 26.470 ms (X-axis), and 24.338 ms (Y-axis). The small dispersion in these results indicates the signal’s phase stability and the precision of pulse-front detection. The highest time delay value was recorded between sensor FOS6-02 and the most distant sensor FOS6-01 in this configuration, where, for the X-axis, the difference of signal time arrival was equal to 86.144 ms. This delay distribution is fully inherent to the spatial architecture of the measurement network and confirms the correctness of the geometric model, assuming wave propagation from explosion point no. 2 towards the remaining array elements. In the dynamic analysis, sensor FOS6-02 exhibited the highest absolute amplitude values in all measured directions, reaching, respectively, 1.50 mrad/s (X-axis), 2.39 mrad/s (Y-axis), and a dominant component of 4.52 mrad/s (Z-axis). The fact that the sensor with the shortest response time recorded the highest signal energy directly confirms wave geometric scattering and legitimizes sensor FOS6-02 as being in the direct zone of influence.

**Table 3:** Parameters of the recorded signals during explosion no. 2 for particular axis of the sensors FOS6-01/02/03/04.

FOS6	Axis	Arrival of the event signal [h: min: s]	End of the event signal [h: min: s]	Event duration time [ms]	Time difference of the event signal arrival [ms] Δt_FOS6−01/−02_	A_max_ [mrad/s]
FOS6-01	Z	12:29:23.643321	12:29:24.053581	209.784	86.144	0.15
	X	12:29:23.638881	12:29:24.125509	206.624	80.029	0.13
	Y	12:29:23.605136	12:29:24.121069	222.419	46.284	0.25
					Δt_FOS6−03/−01_	
FOS6-02	Z	12:29:23.557177	12:29:23.705880	148.703	63.119	1.50
	X	12:29:23.558852	12:29:23.734013	175.161	60.802	2.39
	Y	12:29:23.558852	12:29:23.741716	182.864	21.140	4.52
					Δt_FOS6−01/−04_	
FOS6-03	Z	12:29:23.619012	12:29:23.888478	269.466	22.815	0.12
	X	12:29:23.620296	12:29:23.876288	255.992	26.470	0.60
	Y	12:29:23.619654	12:29:23.917349	297.695	24.338	0.32
					Δt_FOS6−03/−02_	
FOS6-04	Z	12:29:23.579992	12:29:23.763904	183.912	61.835	0.88
	X	12:29:23.585322	12:29:23.846532	261.210	61.444	0.55
	Y	12:29:23.583190	12:29:23.783628	200.438	60.802	1.04

In the third experimental series, the explosion epicenter was located in the interstitial space, at the geometric center between sensors FOS6-02 and FOS6-04 (Fig. 5a). This configuration enabled a critical test of the system’s time-synchronization precision and verification of the array’s sensitivity under quasi-symmetric conditions.

Data obtained from signal acquisition (Table 4) showed almost simultaneous recording of the shock wave front by both sensors in all three orthogonal axes (X, Y, Z). Particularly noteworthy is the fact that the maximum recorded difference in the signal time arrival was only 857 µs for the Z axis. Such a low level of differential error, in the order of microseconds, indicates the exceptional stability of the measurement paths and the almost perfect, symmetrical distribution of the wave propagation path from the source to the sensors.

In the amplitude analysis area, sensor FOS6-02 recorded the highest absolute amplitude value of 3.99 mrad/s for the X-axis. Despite similar wave arrival times, the slight dominance of the amplitude on sensor FOS6-02 suggests local differentiation of propagation conditions or a minimal shift of the explosion energy vector towards this node, which allows for very precise determination of the location of the point inside the array.

**Table 4:** Parameters of the recorded signals during explosion no. 3 for particular axis of the sensors FOS6-01/02/03/04.

FOS6	Axis	Arrival of the event signal [h: min: s]	End of the event signal [h: min: s]	Event duration time [ms]	Time difference of the event signal arrival [ms] Δt_FOS6−01/−02_	A_max_ [mrad/s]
FOS6-01	Z	12:32:21.039424	12:32:21.278168	209.784	89.644	0.07
	X	12:32:21.040301	12:32:21.311497	206.624	89.443	0.08
	Y	12:32:21.042056	12:32:21.380788	222.419	91.737	0.09
					Δt_FOS6−03/−01_	
FOS6-02	Z	12:32:20.949780	12:32:21.164703	214.923	53.867	0.52
	X	12:32:20.950858	12:32:21.131845	180.987	51.946	3.99
	Y	12:32:20.950319	12:32:21.129152	178.833	55.297	2.26
					Δt_FOS6−02/−04_	
FOS6-03	Z	12:32:21.003647	12:32:21.242651	239.004	0.857	0.13
	X	12:32:21.002804	12:32:21.219594	216.790	0.221	0.25
	Y	12:32:21.005616	12:32:21.245182	239.566	0.291	0.18
					Δt_FOS6−03/−02_	
FOS6-04	Z	12:32:20.950637	12:32:21.090410	139.773	0.054	1.85
	X	12:32:20.950637	12:32:21.088175	137.538	0.052	1.95
	Y	12:32:20.950028	12:32:21.084721	134.693	0.055	2.15

The location of the fourth explosion was determined to be near sensor FOS6-04 (Fig. 6a). Due to the short distance between sensors FOS6-04 and FOS6-02 (approx. 12 m), the signals were recorded almost simultaneously (Table 5). However, thanks to precise time synchronization, it was possible to determine the differences in signal arrival time between the two sensors, which were 8.29 ms (Z-axis), 4.36 ms (X-axis), and 11.13 ms (Y-axis). In contrast, the time difference of arrival measurements between the most distant sensors, FOS6-04 and FOS6-01, showed significantly higher values: 104.79 ms (Z-axis), 99.70 ms (X-axis), 105.79 ms (Y-axis). Analysis of recorded signals parameters during explosion no. 4 demonstrated a direct correlation between the geometric distance of the recording FOS6s and the energy of the vibration signal. The highest absolute signal amplitude value was recorded by FOS6-04 (0.70 mrad/s, 0.79 mrad/s, and 0.97 mrad/s for Z, X, and Y axis, respectively, Table 5), which, according to the laws of wave propagation in a continuum medium, confirms its shortest distance from the event’s epicenter. This phenomenon results directly from minimizing energy losses caused by geometric attenuation and energy dissipation within the medium. The opposite pole of the characteristic is represented by the FOS6-01, which recorded the lowest signal amplitude (0.09 mrad/s, 0.13 mrad/s, 0.14 mrad/s for Z, X, Y axes, respectively), which allows for the confirmation of the farthest localization from the explosion source, resulting in the signal being most attenuated before reaching the sensor.

The presented experimental data enable clear quantification of the usefulness of the FOS6s for precisely locating sources of acoustic (or seismic) disturbances. The observed time-of-arrival correlations are consistent with the hypothesis of wave propagation and their geometric spatial distribution. The experiments were conducted in the near-field regime, as evidenced by the high signal intensities and short propagation times. In light of the obtained results, it is postulated that further research should be conducted in a much larger measurement field, which will enable the assessment of signal attenuation characteristics as a function of distance and the verification of the method’s scalability.

Analysis of the signal amplitude recorded by the nearest sensor during four explosions showed that the highest value has been recorded for the Y-axis, [14.01 mrad/s (FOS6-01), 4.52 mrad/s (FOS6-02), 2.15 mrad/s (FOS6-04), 0.97 mrad/s (FOS6-04) for explosions 1–4, respectively].

**Table 5:** Parameters of the recorded signals during explosion no. 4 for particular axis of the sensors FOS6-01/02/03/04.

FOS6	Axis	Arrival of the event signal [h: min: s]	End of the event signal [h: min: s]	Event duration time [ms]	Time difference of the event signal arrival [ms] Δt_FOS6−01/−02_	A_max_ [mrad/s]
FOS6-01	Z	12:57:02.095326	12:57:02.529798	209.784	96.496	0.09
	X	12:57:02.094167	12:57:02.526322	206.624	95.337	0.13
	Y	12:57:02.097643	12:57:02.506626	222.419	94.667	0.14
					Δt_FOS6−04/−01_	
FOS6-02	Z	12:57:01.998830	12:57:02.432092	433.262	104.789	0.11
	X	12:57:01.998830	12:57:02.434165	435.335	99.697	0.38
	Y	12:57:02.002976	12:57:02.427946	424.97	105.795	0.24
					Δt_FOS6−02/−04_	
FOS6-03	Z	12:57:02.064065	12:57:02.469138	405.073	8.293	0.13
	X	12:57:02.067029	12:57:02.500753	433.724	4.360	0.25
	Y	12:57:02.067029	12:57:02.479018	411.989	11.128	0.24
					Δt_FOS6−03/−02_	
FOS6-04	Z	12:57:01.990537	12:57:02.391235	400.698	65.235	0.70
	X	12:57:01.994470	12:57:02.288111	293.641	68.199	0.79
	Y	12:57:01.991848	12:57:02.358463	366.615	64.053	0.97

This energy distribution suggests that the shock wave front generated by the explosion propagated with a dominant directional component in the horizontal plane, exhibiting distinct spatial anisotropy. This suggests that the driving force vector, or the main energy propagation channel, was oriented parallel to the Y axis, which may be due to the geometry of the explosive charge, wave directionality through terrain obstacles, or the characteristics of the source itself. In turn, the lowest amplitude values recorded for the Z-axis [vertical direction, 4.12 mrad/s (FOS6-01), 1.50 mrad/s (FOS6-02), 1.85 mrad/s (FOS6-04), 0.70 mrad/s (FOS6-04) for explosions 1–4, respectively] indicate a relatively small contribution of the vertical component to the total energy balance of the disturbance at the measurement point. This disparity between the Y and Z axes indicates that the shock wave was primarily surface or longitudinal in nature, and that its impact in the vertical plane was more strongly attenuated or was initially limited by the specific mechanism of explosion generation. The obtained results confirm that multiaxial analysis allows for precise determination of the wave propagation vector and identification of the dominant directions of medium displacements, which is crucial for modeling dynamic effects in the near field.

## Conclusion

The presented experimental data confirmed the high effectiveness of the developed detection system based on an array of FOS6 in near filed. The experimental results are fully consistent with the theoretical assumptions, demonstrating that the sensors are sufficiently sensitive to accurately record shock waves with high temporal synchronization. A key achievement of this work is the demonstration of a close correlation between the recorded signals and the event’s actual location, with precise time-stamping. The minimal time differences observed between individual array nodes demonstrate the high precision of the measurement system. The system’s ability to faithfully reconstruct the explosion source’s actual location from time-series data confirms that the proposed disturbance localization method exhibits high spatial resolution and provides a foundation for advanced early-warning systems.

The successful validation of the prototype provides a clear direction for further research. The next necessary step is to conduct tests at greater distances from the signal source. Increasing the measurement base (array span) will enable precise back-azimuth calculations using data only about rotational components, which are crucial for fully identifying the direction of wave propagation and for more accurate triangulation in the field. Further research will also focus on optimizing the algorithms to ensure system scalability across large areas of critical infrastructure.

## Supplementary Information

Below is the link to the electronic supplementary material.

Supplementary Material 1

## Data Availability

The data that support the findings of this study are available on request from the corresponding author A.T.K.
